# Genetically Predicted Pulse Pressure and Risk of Abdominal Aortic Aneurysm: A Mendelian Randomization Analysis

**DOI:** 10.1161/CIRCGEN.121.003575

**Published:** 2022-05-06

**Authors:** Stephen Burgess, Julio A. Chirinos, Scott M. Damrauer, Dipender Gill

**Affiliations:** 1 Medical Research Council Biostatistics Unit, Cambridge Institute of Public Health, United Kingdom (S.B.).; 2 Cardiovascular Epidemiology Unit, Department of Public Health and Primary Care, University of Cambridge, United Kingdom (S.B.).; 3 Division of Cardiovascular Medicine, Hospital of the University of Pennsylvania, University of Pennsylvania Perelman School of Medicine, Philadelphia (J.A.C.).; 4 Departments of Surgery and Genetics, Perelman School of Medicine, University of Pennsylvania, Philadelphia (J.A.C., S.M.D.).; 5 Corporal Michael J. Crescenz VA Medical Center, Philadelphia, PA (S.M.D.).; 6 Department of Epidemiology and Biostatistics, School of Public Health, Imperial College London (D.G.).; 7 Clinical Pharmacology Group, St George’s University Hospitals NHS Foundation Trust (D.G.).; 8 Clinical Pharmacology and Therapeutics Section, Institute for Infection and Immunity, St George’s, University of London (D.G.).; 9 Novo Nordisk Research Centre Oxford, United Kingdom (D.G.).

**Keywords:** aortic aneurysm, blood pressure, genetics, meta-analysis, population

Pulse pressure (PP), the difference between systolic blood pressure (SBP) and diastolic blood pressure (DBP), arises due to pulsatile ejection of blood from the left ventricle. Previous observational studies have identified an inverse association of PP with aortic diameter,^1^ and positive associations of PP with aortic wall stiffness and thickness.^2^ However, it is not known whether these associations reflect a causal effect of PP on the risk of abdominal aortic aneurysm (AAA), an effect of the aorta on PP, a shared cause, or confounding from environmental factors.

Here, we investigated the relationship between PP and AAA risk using 2-sample Mendelian randomization, which employs genetic variants specifically related to an exposure to define subgroups of the population with different average levels of the exposure. The independent segregation of alleles at conception means these genetically defined subgroups should not differ systematically with respect to confounding variables, creating a natural experiment analogous to a randomized trial.

First, to investigate the relationship between PP and risk of AAA independent of other BP measures, we performed multivariable Mendelian randomization using the inverse-variance weighted method.^3^ In our main analysis, PP and mean arterial pressure are the exposures and AAA is the outcome. In secondary analyses, we consider SBP and DBP respectively with PP as exposures in multivariable analyses. AAA events were identified in UK Biobank, a population-based cohort of UK residents aged 40 to 69 at baseline, based on electronic heath records. Genetic associations with AAA were estimated in 367 586 European ancestry participants (1094 AAA cases) by logistic regression adjusting for age, sex, and 10 principal components of genetic ancestry (to account for potential population stratification). As genetic instruments for BP traits, we selected 258 uncorrelated variants previously associated with BP at a genome-wide level of significance in the International Consortium for Blood Pressure^4^ excluding UK Biobank participants. Genetic associations with BP measures were obtained by meta-analysis of study-specific estimates estimated using linear regression in 299 024 European ancestry participants from the International Consortium for Blood Pressure with UK Biobank participants excluded.

Second, to explore the association of genetically predicted AAA risk with BP measurements, we performed univariable Mendelian randomization analyses with AAA risk as the exposure and BP measures as the outcome. This investigates the relationship between liability to AAA and BP.^3^ As instruments for AAA, we considered 24 uncorrelated variants associated with AAA risk at a genome-wide level of significance.^5^ For these analyses, genetic associations with BP measures were estimated in UK Biobank using linear regression adjusting for age, sex, and 10 principal components.

All data are publicly available at http://dx.doi.org/10.6084/m9.figshare.17912192. UK Biobank has approval from the North West Multicentre Research Ethics Committee.

Considering the effect of BP on AAA, genetically predicted mean arterial pressure was positively associated with AAA risk (estimates are scaled to a 5 mm Hg increase in the BP trait; odds ratio [OR], 1.55 [95% CI, 1.21–2.00]; *P*=0.008), whereas genetically predicted PP was inversely associated (odds ratio, 0.64 [95% CI, 0.46–0.89]; *P*=0.0006). Similar findings were observed in multivariable analyses for SBP and PP, and DBP and PP (Figure). In sex-stratified analyses, inverse associations with PP were similar in magnitude for males and females, although less precise in females with 95% CIs overlapping the null (Figure).

**Figure.**
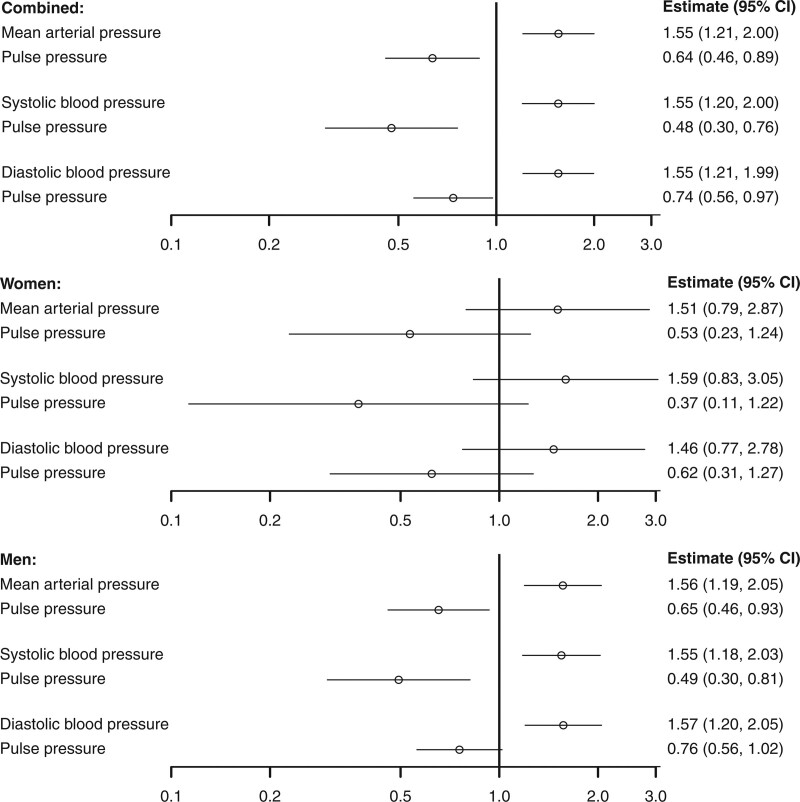
**Associations between genetically predicted blood pressure measures and abdominal aortic aneurysm from 3 separate multivariable Mendelian randomization analyses using combined and sex-stratified genetic associations with abdominal aortic aneurysm risk.** Estimates (95% CI) represent the odds ratio for disease per 5 mm Hg increase in genetically predicted levels of the blood pressure trait. Separate multivariable analyses were performed for 3 choices of exposure variables: mean arterial pressure and pulse pressure; systolic blood pressure and pulse pressure; and diastolic blood pressure and pulse pressure. Abdominal aortic aneurysm (AAA) was defined from hospital episode statistics and death certificates using *International Classification of Disease* coding (*Ninth Edition*: 441.3 or 441.4, *Tenth Edition* I71.3 or I71.4) or hospital procedure coding (Office of Population Censuses and Surveys code: L19.4 or L19.5).

Considering the effect of AAA risk on BP measures, genetically predicted AAA risk was inversely associated with PP, with a 0.19 mm Hg (95% CI, 0.08–0.29; *P*=0.0006) reduction per unit increase in the log-odds of AAA. An inverse association was also observed with SBP (0.17 mm Hg [95% CI, 0.02–0.32]; *P*=0.024) but not with DBP (−0.01 mm Hg [95% CI, −0.10 to 0.07]; *P*=0.76) or mean arterial pressure (0.05 mm Hg [95% CI, −0.05 to 0.15]; *P*=0.34).

This Mendelian randomization study advances on previous epidemiological investigations to provide evidence supporting a bidirectional inverse relationship between PP and AAA risk, consistent with a shared cause. Increased stiffness of the aortic wall may underlie this, by raising PP (due to higher SBP and lower DBP) but decreasing risk of AAA (due to less distension of the aortic wall). Another explanation is a threshold effect for case classification, where AAA classification is less likely in individuals with smaller aortas because diagnosis is based on absolute rather than relative diameter. However, estimates were similar in males and females, despite sex differences in aorta size. Limitations of our investigation are that we did not have data on lumen diameter, were not able to assess specific AAA causes, and findings may not relate equally to all AAA subtypes. A further limitation is power, particularly for the female-specific analysis.

Our study provides important mechanistic insights about the relationship between PP and the risk of AAA. It suggests an inverse relationship between PP and AAA risk but one that is likely driven by a common underlying mechanism rather than a direct inverse causal effect of PP on AAA risk.

## Article Information

### Acknowledgments

S. Burgess had full access to all the data in the study and takes responsibility for the integrity of the data and the accuracy of the data analysis. This research has been conducted using the UK Biobank resource (application 29202). The UK Biobank data is available on application (http://www.ukbiobank.ac.uk/register-apply).

### Sources of Funding

S. Burgess is supported by Sir Henry Dale Fellowship jointly funded by the Wellcome Trust and the Royal Society (204623/Z/16/Z). Dr Chirinos is supported by National Institutes of Health (NIH) grants R01-HL 121510, R33-HL-146390, R01HL153646, R01-AG058969, 1R01-HL104106, P01-HL094307, R03-HL146874, and R56-HL136730. Dr Damrauer is supported by the US Department of Veterans Affairs (IK2-CX001780). This publication does not represent the views of the Department of Veterans Affairs or the United States Government. D. Gill is supported by the British Heart Foundation Centre of Research Excellence (RE/18/4/34215) at Imperial College London and a National Institute for Health Research Clinical Lectureship at St George’s, University of London (CL-2020-16-001). This research was supported by the National Institute of Health Research Cambridge Biomedical Research Centre (BRC-1215-20014). The views expressed are those of the author(s) and not necessarily those of the National Institute of Health Research or the Department of Health and Social Care.

### Disclosures

Dr Chirinos has recently consulted for Bayer, Sanifit, Fukuda-Denshi, Bristol-Myers Squibb, JNJ, Edwards Life Sciences, Merck and the Galway-Mayo Institute of Technology. Dr Chirinos is supported by National Institutes of Health (NIH) grants R01-HL 121510, R33-HL-146390, R01HL153646, R01-AG058969, 1R01-HL104106, P01-HL094307, R03-HL146874, and R56-HL136730. He is named as inventor in a University of Pennsylvania patent for the use of inorganic nitrates/nitrites in Heart Failure with Preserved Ejection Fraction. He has received research device loans from Atcor Medical, Fukuda-Denshi, Uscom, NDD Medical Technologies, Microsoft and MicroVision Medical. S.M. Damrauer has received grants from the US Department of Veterans Affairs, Calico Labs, and Renalytix AI outside the submitted work. D. Gill is employed part-time by Novo Nordisk and has received consultancy fees from Policy Wisdom. The other author reports no conflicts.
